# TDS Similarity: Outlier Analysis Using a Similarity Index to Compare Time-Series Responses of Temporal Dominance of Sensations Tasks

**DOI:** 10.3390/foods12102025

**Published:** 2023-05-17

**Authors:** Hiroharu Natsume, Shogo Okamoto, Hikaru Nagano

**Affiliations:** 1 Department of Computer Science, Tokyo Metropolitan University, Hino 191-0065, Japan; natsume-hiroharu@ed.tmu.ac.jp; 2 Department of Mechanical Engineering, Kobe University, Kobe 657-8501, Japan; nagano@mech.kobe-u.ac.jp

**Keywords:** temporal dominance of sensations, individual analysis, similarity index, discrete time, strawberry

## Abstract

Temporal dominance of sensations (TDS) methods are used to record temporally developing sensations while eating food samples. Results of TDS tasks are typically discussed using averages across multiple trials and panels, and few methods have been developed to analyze differences between individual trials. We defined a similarity index between two time-series responses of TDS tasks. This index adopts a dynamic level to determine the importance of the timing of attribute selection. With a small dynamic level, the index focuses on the duration for attributes to be selected rather than on the timing of the attribute selection. With a large dynamic level, the index focuses on the temporal similarity between two TDS tasks. We performed an outlier analysis based on the developed similarity index using the results of TDS tasks performed in an earlier study. Certain samples were categorized as outliers irrespective of the dynamic level, whereas the categorization of a few samples depended on the level. The similarity index developed in this study achieved individual analyses of TDS tasks, including outlier detection, and adds new analysis techniques to TDS methods.

## 1. Introduction

The temporal dominance of sensations (TDS) method [1,2] enables us to record the temporal evolution of multiple types of sensations, including gustatory, olfactory, and textural feelings while eating food samples. Such time evolution is caused by the physical, chemical, and thermal changes of foods in the mouth, part of which is brought about by saliva [3] and mouth and tongue motion [4], temporal changes in sensory responses including adaptation at peripheral and central nervous levels [5], and cross-modal interactions [6,7,8,9]. For example, the habituation of olfactory sensations is prominent in humans [5,10] and may affect the time-series responses while eating foods. TDS methods have garnered attention in food science since the 2010s because they are more cost-effective than previous time-series sensory evaluation methods such as the time-intensity method [11,12]. The properties of the data acquired through TDS methods and analysis techniques for TDS methods continue to be intensively studied by many researchers [13,14,15]. TDS methods are largely used for food and beverages; however, recent studies have applied the method to other types of stimuli and modalities. Examples include audio [16], visual [17], audiovisual [18], and haptic stimuli [19,20]. Furthermore, there exist modified TDS methods, including the temporal check-all-that-apply method [21,22]. In addition, the TDS method can be used to evaluate temporal and overall food liking or preferences [23,24] and to investigate their temporal drivers.

The TDS method is primarily used to determine the tendency of entire panels, and the average of multiple trials from all panels is often discussed. For example, the TDS curve [2], a typical analysis and visualization method for the TDS method, plots the proportion of the panels that select each sensory attribute as dominant over time. Such proportions are called dominance proportions. Inevitably, other analysis methods based on TDS curves, including sensory trajectories [25] and casual analyses [26,27], deal with the average response among the panels as well. These average responses are influenced by outlier samples; however, methods to detect outliers have not been developed for TDS methods. Meyners et al. proposed a method to test whether a panel is statistically different from the other panels using a randomization test [28]. Lepage et al. developed a method to test whether panels differ from each other in their sensory responses and abilities to distinguish food products based on dominance durations that are time periods for descriptive attributes to be selected as dominant [29]. These methods assume that a panel repeatedly evaluates the same foods. Therefore, the methods developed by Meyners [28] and Lepage et al. [29] cannot be applied to individual TDS trials. It is possible to simulate time-series responses of TDS tasks with semi-Markov chains [30,31,32]. Cardot et al. [30] classified the whole panel into several semi-Markov processes according to the characteristics of their responses. However, this corresponded to a classification analysis of panels, and was not intended to discuss individual TDS trials. Individual differences and effects of mental states exist in food preferences and perception [33,34,35,36,37]; hence, a method for analyzing differences among multiple TDS trials is required. However, to date no method has been proposed to investigate the similarity or dissimilarity between different TDS trials.

Owing to the quantitative similarities or dissimilarities between multiple TDS trials, several analytical methods that require distance scales between samples can be developed. Representative examples of such methods include outlier analysis and hierarchical clustering. In this study, we developed an index to evaluate the similarity between two TDS trials. This index compares the time series responses of two trials discretized into *R* intervals using the Euclidean distance. The value of *R* is the level of dynamics, and indicates the extent to which the temporal aspect should be considered necessary. For small *R* values, the focus is on the similarity of attribute selection during the trial periods between two trials rather than on the temporal similarity between the trials. We applied this index to the result of TDS tasks on strawberries [38] and examined how the similarity index depends on *R*.

As an essential application of the similarity index, we performed an outlier analysis of TDS tasks for strawberries. To the best of our knowledge, outlier analysis methods have not yet been developed for TDS methods. Even well-controlled experiments cannot avoid outliers in samples. Our study can provide the TDS method with a common data-preprocessing method to detect outliers. The present study is an extension of an earlier study [39] in which the concept of dissimilarity between TDS trials was introduced; however, the variable *R* was not used. In the present study, the effects of the *R* values are intensively investigated.

## 2. TDS Method and Task

**Figure 1:**
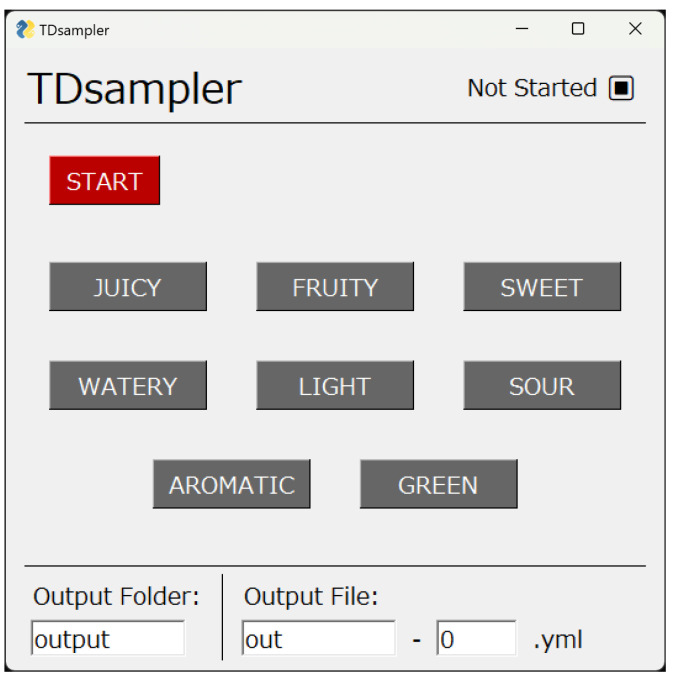
Graphical user interface used for recording TDS tasks. In the software shown here, the START button turns into the STOP button after it is pressed.

In this section, the typical task of the TDS method and the calculation of dominance proportions are introduced. More information can be acquired from [1,13]. As shown in Figure 1, the tasks of the TDS method are performed using a graphical user interface on a computer. The interface has a maximum of 10–12 buttons with labels of attribute words representing tastes, odors, and mouth feels. In addition, there are two buttons to start and stop the recording. When a panel performs a task, the participant places the food sample in their mouth and simultaneously presses the start button. The panel then selects buttons with attribute words. These buttons correspond to the most dominant sensation that the panel experiences at each moment. Multiple buttons cannot be selected at the same time. After selecting one attribute button, that button is kept selected until another button is selected. The same attribute words can be selected more than one time or never. When the food is swallowed, the panel presses the stop button, and the task is terminated. The computer application records the time when the panel selects the buttons. Individual panels repeat the tasks for the same food sample several times.

**Figure 2:**
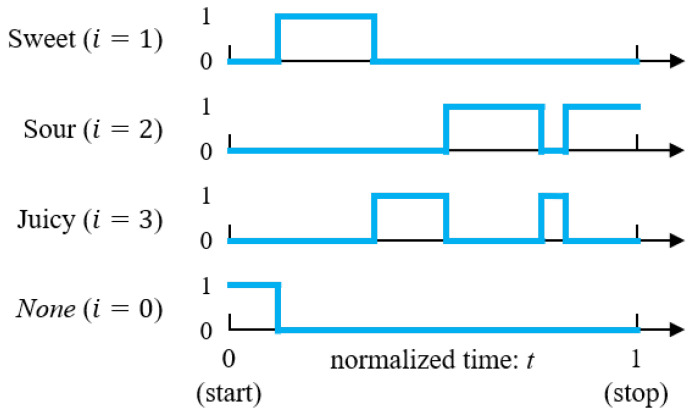
Result of a TDS task as binary functions $x_{i}^{\left(j\right)}\left(t\right)$. None is 1 only when no attribute is selected immediately after the start button is pressed. At any moment, only one attribute takes the value 1.

The result of an example TDS task is shown in Figure 2. The response to attribute *i* in the *j*th TDS trial is expressed by a binary function $x_{i}^{\left(j\right)}\left(t\right)\in \left\{0,1\right\}$ of continuous time *t*. When attribute *i* is selected at *t* in *j*th trial, $x_{i}^{\left(j\right)}\left(t\right)=1$; otherwise, $x_{i}^{\left(j\right)}\left(t\right)=0$. The continuous time *t* is the normalized time ranging from 0 to 1. The commencement and end of the individual task correspond to t=0 and t=1, respectively.

We use an additional function $x_{0}^{\left(j\right)}\left(t\right)\in \left\{0,1\right\}$, which equals 1 only before the first attribute button is selected. In other words, after a certain button has been selected, it remains 0 until the end. Hence, at each moment *t*, the following equation holds

(1) $$\begin{array}{c} \sum_{i=0}^{q}x_{i}^{\left(j\right)}\left(t\right)=1, \end{array}$$

where *q* is the number of attributes used in the TDS task. Multiple trials are averaged using the following equation:

(2) $$\begin{array}{c} p_{i}\left(t\right)=\frac{1}{n}\sum_{j=1}^{n}x_{i}^{\left(j\right)}\left(t\right), \end{array}$$

where *n* is the number of trials (the number of panels × repetitions) and $p_{i}\left(t\right)$ represents the proportion with which attribute *i* is selected at *t* among all the trials. After being smoothed over time, this proportion is referred to as a dominance proportion [1].

## 3. Similarity Index for TDS Trials

A method for calculating the similarity of the results of two arbitrary TDS tasks is described in this section. The results of the TDS tasks, which are continuous functions, are converted to discrete functions before computing the similarity. This process follows

(3) $$\begin{array}{c} X_{i}^{\left(j\right)}\left[k\right]=R\int_{k/R}^{(k+1)/R}x_{i}^{\left(j\right)}\left(t\right)dt, \end{array}$$

where *R* is the number of discretized time intervals and $X_{i}^{\left(j\right)}\left[k\right]$ is the average value of $x_{i}^{\left(j\right)}$ on the *k*th discretization interval (k=0,1,…,R−1), is equivalent to the dominance duration in the *k*th interval. The interval used to calculate the dominance durations is 1/R. When R=1, $X_{i}^{\left(j\right)}\left[k\right]$ has the value only for k=0, which represents the dominance duration for the entire period of the trial. This conversion from continuous to discrete forms can be applied to the dominance proportion $p_{i}\left(t\right)$ as well, which is expressed as $P_{i}\left[k\right]$.

We defined the similarity index *S* between two TDS trials *a* and *b* with a certain *R* value:

(4) $$\begin{array}{c} S_{R}\left(X^{\left(a\right)},X^{\left(b\right)}\right)=1-\frac{1}{\sqrt{2}R}\sum_{k=0}^{R-1}\sqrt{\sum_{i=0}^{q}{\left|X_{i}^{\left(a\right)}\left[k\right]-X_{i}^{\left(b\right)}\left[k\right]\right|}^{2}}. \end{array}$$

The second term of (4) corresponds to the average Euclidean distance in a (q+1)-dimensional space between TDS trials *a* and *b*, which is normalized by $\sqrt{2}R$ such that its maximum value is 1. In the (q+1)-dimensional space, the coordinates of the *i*th axis are $X_{i}^{\left(a\right)}\left[k\right]$ and $X_{i}^{\left(b\right)}\left[k\right]$. The distance corresponds to the dissimilarity of the two trials. The similarity is the complement of the dissimilarity for the 1.

In addition, $S_{R}$ can be applied to the dominance proportion *P*. For example, $S_{R}\left(X^{\left(a\right)},P\right)$ indicates the similarity of trial $X^{\left(a\right)}$ to the centroid of entire trials. We defined the similarity between the two trials; however, the dissimilarity or distance in the second term of (4) may be preferable for different analysis purposes. Certain aspects of the above computational process are similar to the method proposed by Pineau et al. for hypothesis testing of two sets of TDS curves [13].

## 4. TDS Data of Strawberries

We used the data from TDS tasks collected by Shimaoka et al. [38]. They conducted the TDS tasks for strawberries by involving seventeen paid university student panels (fourteen males and three females) in their 20s. They were not experts in the sensory evaluation of foods, and familiarized themselves with the TDS tasks, including how to use graphical user interfaces, before the experiment for several minutes until they felt confident. Individual panels replicated three TDS tasks in which one strawberry was eaten in each task. In total, 51 tasks were conducted (n=51). The participants used eight types of attributes (q=8) selected by ten participants through a vote among 218 candidate attributes. The eight attributes were *sweet*, *sour*, *fruity*, *green*, *watery*, *juicy*, *aromatic*, and *light*.

**Figure 3:**
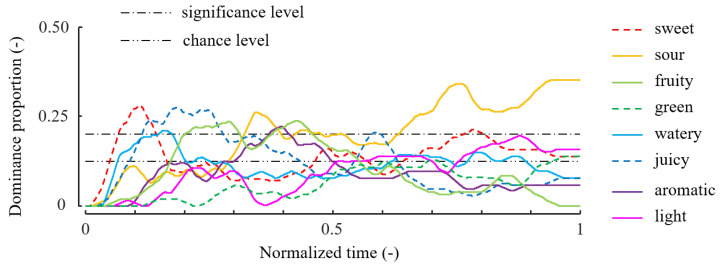
TDS curve: time-series of the average of all trials. This curve is called the TDS curve. The dominance proportion (the vertical axis) is the proportion of trials in which an attribute word was selected at a given time. Adapted from [38].

Figure 3 shows the dominance proportions calculated from all the tasks. Typically, in the earlier phase, *sweet*, *juicy*, and *fruity* were dominantly selected. The attribute *aromatic* was more prominent in the middle phase than in the other phases. Further, *sour* was frequently selected in the middle phase, and remained dominant in the last period. Similar results were reported in another study of TDS tasks with strawberries [26].

## 5. Similarity Index with Different *R* Values: Example of Strawberries

The similarity index varies with *R*. In particular, the index is almost different between small *R* values (such as 1–3) and large *R* values (such as 100). Here, using strawberries as an example, we investigated how the similarity indices are different between different *R* values. We calculated the correlation coefficients between the similarity index values between different *R* values to obtain the influences of *R* values on similarity scores.

We assumed the strawberry data in the previous section to be a set of continuous functions for *t*: $\left\{x_{i}^{\left(j\right)}\left(t\right)|j=1,2,\ldots ,51;i=0,1,\ldots ,8\right\}$, where 51 and 8 are the numbers of TDS tasks or replications and attributes, as used in the previous experiment [38]. As in (2), $p_{i}\left(t\right)$ is the average of all trials or dominance proportions. For given values of *R*, $C_{R}$ is the set of the values of $S_{R}$ for each trial and $p_{i}\left(t\right)$:

(5) $$\begin{array}{c} C_{R}=\left\{S_{R}\left(X^{\left(j\right)},P\right)|j=1,2,\ldots ,51\right\}, \end{array}$$

where $X^{\left(j\right)}$ and *P* are the symbols of the *j*th TDS trial and mean dominance proportions, respectively. When R=1, the dominance durations of two trials were compared without considering the temporality. When R=3, the similarity was computed for each of the three intervals, that is, the early, middle, and last phases of the task, and the temporality of the dominance proportions was moderately considered. When R=10, provided that the average eating period of a strawberry was about 30 s [38], two pairs of trials were compared at intervals of approximately 3 s. The correlation coefficient between two sets $C_{R1}$ and $C_{R2}$ is defined as

(6) $$\begin{array}{c} r_{R1,R2}=\frac{\sum_{j=1}^{51}\left(C_{R1}^{\left(j\right)}-{\bar{C}}_{R1}\right)\left(C_{R2}^{\left(j\right)}-{\bar{C}}_{R2}\right)}{\sigma_{R1}\sigma_{R2}} \end{array}$$

where $\sigma_{R1}$ and $\sigma_{R2}$ are the standard deviations of $C_{R1}$ and $C_{R2}$, respectively, and ${\bar{C}}_{R1}$ and ${\bar{C}}_{R2}$ are the mean values of $C_{R1}$ and $C_{R2}$, respectively.

**Figure 4:**
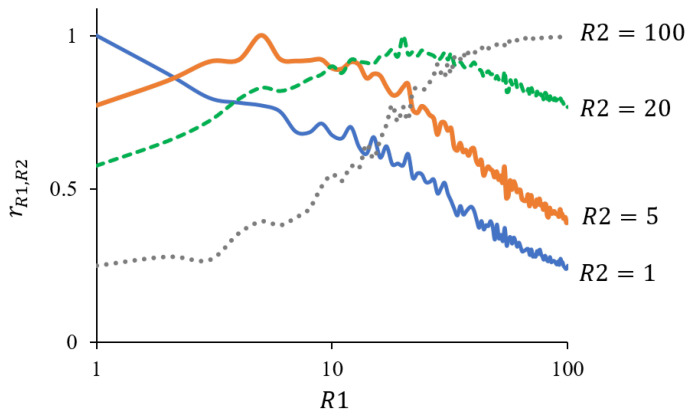
$r_{R1,R2}$: Correlation coefficient of similarities between $C_{R1}$ and $C_{R2}$. The numbers of the discretized intervals are R1={1,2,…,100} and R2={1,5,20,100}.

The $r_{R1,R2}$ values for $\left(R1,R2\right)\in {\left\{1,3,10,30,50,100\right\}}^{2}$ are shown in Table 1. When the values of R1 and R2 were close, the two sets $C_{R1}$ and $C_{R2}$ are correlated more strongly. For example, the indices for R1=1 and R2=3 are strongly correlated with $r_{1,3}=0.797$; however, those for R1=1 and R2=100 are only weakly correlated with $r_{1,100}=0.251$. Figure 4 is the graphical version of Table 1, and shows $r_{R1,R2}$ values with $R2=\left\{1,5,20,100\right\}$ and R1 varying from 1 to 100. Similar to Table 1, the correlation coefficient between $C_{R1}$ and $C_{R2}$ is higher when R1 and R2 are closer to each other. When R2=20, the correlation coefficient is greater than 0.5 for any R1, indicating that the similarity values for R=20 are largely or moderately correlated with those for the other *R* values.

## 6. Outlier Detection

### 6.1. Methods

To test the normality of the value distribution in $C_{R}$, we applied the Kolmogorov–Smirnov normality test to $C_{R}$. When the hypothesized normality was not denied, we judged potential outliers using the range defined by the mean and standard deviation. Trials, that is, members in $C_{R}$, were considered potential outliers if they did not fall within the range of [μ−1.64σ,+∞], where μ is the sample mean and σ is the sample standard deviation calculated among all the values in $C_{R}$. This range contains 95% of all the samples, the remaining 5% with small similarity values being screened out. The range was [μ−2.33σ,+∞] for considering 99% of the samples. Note that the upper side of the distribution does not need to be considered because trials with high similarity represent the population well. When the samples in $C_{R}$ are judged to not be subjected to the normal distribution, outlier detection methods should be selected depending on the type of distribution. Typically, a method using box plots and interquartile range can be used for determining outliers among non-normally distributed samples.

If the mean is calculated across all samples, it can be biased by potential outliers. To avoid such cases, a method based on the minimum covariance determinant [40] may be used.

### 6.2. Example of Strawberries

**Figure 5:**
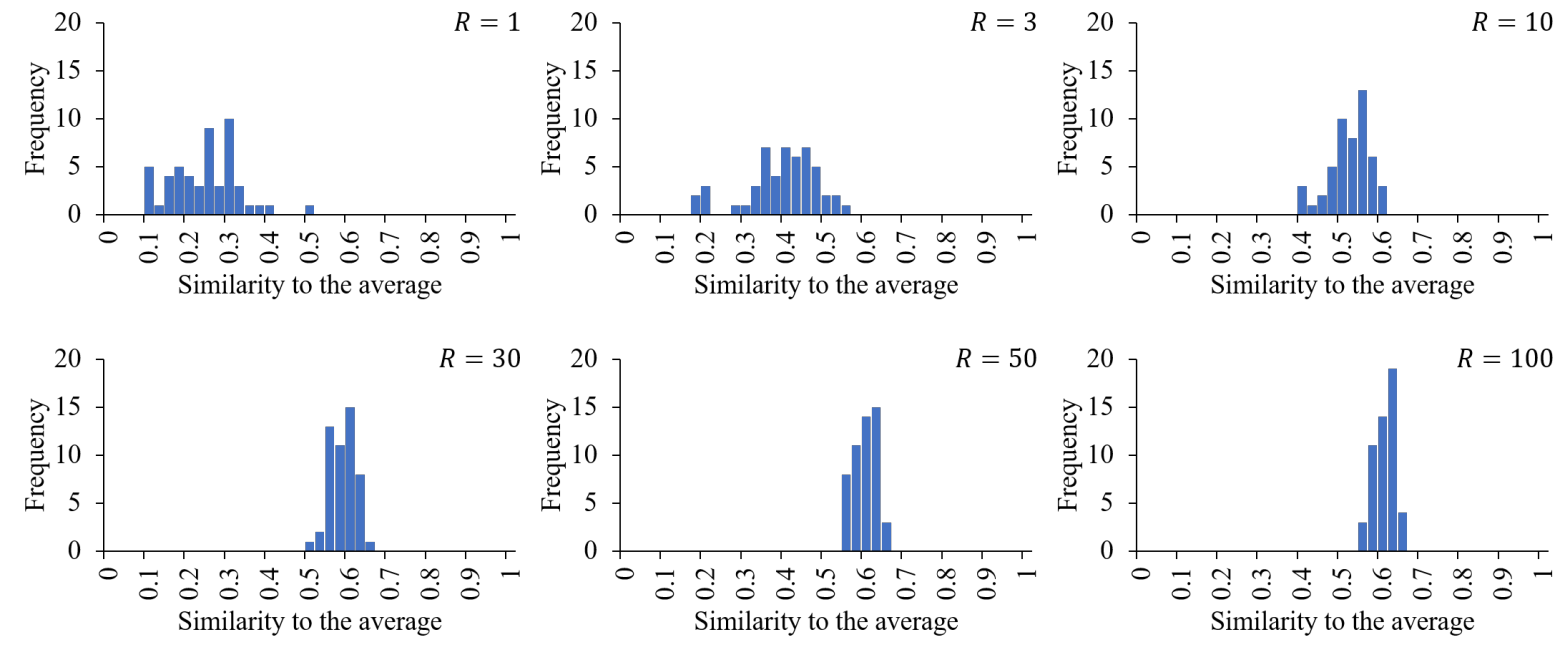
Distribution of similarities between individual trials and the average (*P*) for R=1,3,10,30,50, and 100. The width of each bin is 0.025. For all *R* values, the Kolmogorov–Smirnov test did not reject normality with p<0.05.

**Figure 6:**
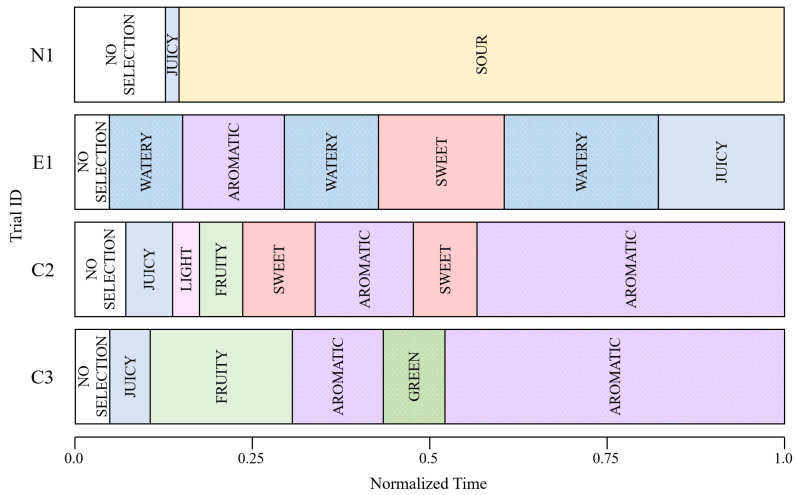
Time-series of potential outliers. Each box with an attribute label represents the duration and timing for which the attribute was selected. “NO SELECTION” means that no attribute was selected during these periods.

We performed an outlier analysis on the TDS data for strawberries for each $R=\left\{1,3,10,30,50,100\right\}$. Figure 5 shows the distribution of the similarity indices between the individual trials and the average, that is, the TDS curves, for each *R* value. The *p*-value of the Kolmogorov–Smirnov test was greater than 0.05 for all *R* values; hence, we assumed the normal distribution to detect potential outliers. The detected outliers are shown in Table 2 as “+”. Each trial is identified by the panel ID of an alphabetic letter and the iteration count of the task. Trial C3, the third trial of panel C, was classified as an outlier for any *R* value, while other trials were classified as such only for small, large, or medium *R* values. For example, N1, that is, the first trial of panel N, was categorized as a potential outlier only when R=1. The time series for each trial are shown in Figure 6.

### 6.3. Discussion: Semantic Validity of Outliers

Here, we discuss or interpret the reasons for certain trials being judged as outliers in the analysis in Section 6.2, which corresponds to the semantic validation of the outlier analysis.

In trial N1, sour was selected for almost the complete duration of the task. As shown in Figure 3, on average sour was the most dominant attribute in the second half of the task. Nevertheless, N1 was classified as an outlier when R=1. This was because the number of attributes selected during the task was too small in trial N1. On average, several types of attributes, such as sweet, juicy, watery, and sour, were selected for sustained periods; however, in trial N1 the dominance durations of all attributes except for sour and juicy were 0, which distinguishes this trial from the average trial.

E1 was classified as an outlier at medium *R* values. In this trial, attributes that exhibited low proportions in Figure 3 were selected over the entire period, such as watery and aromatic. In average trials, watery was largely selected in the early phase of the task, as shown in Figure 3. However, in trial E1 watery was selected even in the middle and last parts of the task. This trial was not considered an outlier when focusing on more chronological details with large *R* values, such as 50 and 100.

In attribute selection, C2 and C3 exhibited similar patterns. However, C2 was classified as an outlier only when R=50, whereas C3 was considered an outlier for every *R* value, as shown in Table 2. For R=30 and 100, C2 exhibited small similarity values; however, they were barely within the range of the inliers. In both trials, aromatic was selected in the later period, in which aromatic was not prominent on average. In the early period, however, typical attributes such as juicy and fruity were selected in both C2 and C3. In C3, juicy and fruity were selected earlier than in the average trial, as shown in Figure 3. Hence, C3 was judged as an outlier with large *R* values, with which the selected attributes were chronically compared. Further, in C3, sour, which was selected in the majority of the trials, was not selected at all. This caused C3 to be judged as an outlier with small *R*, in which dominance durations are more important than timing.

As aforementioned, four trials that were detected as outliers with at least one *R* value seem distinct from the typical panel behavior shown in Figure 3. In the above outlier analysis, different values of *R* led to different outliers. This means that the developed similarity index changes the level of dynamics considered depending on the *R* value.

## 7. General Discussion

A key feature of the developed similarity index is that it can change the degree of emphasis on timing depending on the value of *R*. We assume that an appropriate *R* value depends on the research target, which varies according to the purpose of the analysis and the type of foods to be tested. For example, a low *R* value may be used for foods with large individual differences in eating time, such as chewy foods. For such foods, closely focusing on time-series is not meaningful. In contrast, a large *R* value may be preferred if researchers wish to conduct a detailed investigation of the temporal changes of sensations. As a systematic method, we propose setting *R* based on the correlation coefficients of the similarity values between the different *R* values. In the example of strawberries, as shown in Figure 4, the correlation coefficients between the index values at R=20 and those at the other *R* values ranging from 1–100 exhibit constant high values. Such *R* (in this case, 20) is recommendable because it is in accordance with both small *R* values, where the timings of attribute selection are not deemed important, and large *R* values, where the timings are considered important. For strawberries, the average task duration was approximately 30 s [38]. Hence, for R=20, one interval corresponds to about 1.5 s. Previous studies have discussed TDS curves by dividing the entire trial period into three or four equal parts; in other words, they used R=3 [13,29] or R=4 [41]. However, these studies not provide suggestions on the question of how many intervals the entire period should be split into. One notable criterion for determining the *R* value was suggested in [26], where continuous TDS curves were discretized with an interval of approximately 1 s for causality analysis. The authors referred to the minimum interval between two successive button selections during the TDS tasks. The panels largely switched attributes in more than 1 s. This can be a reasonable method for determining *R*. It may not be beneficial to discuss and analyze the behaviors of panels when the temporal resolution is smaller than the minimum behavioral response time. Another perspective on how to set an appropriate *R* value involves dummy outliers. For example, in our case several TDS samples of non-strawberry foods could have been mixed with those for strawberries. The samples of non-strawberry foods could then be perfectly determined as outliers, with the dummy TDS samples used for performance evaluation of the outlier analysis.

In this study, only strawberries were used as food samples. Therefore, at present the nature of the index discussed above only holds for strawberries. Furthermore, the data used in this study were collected from panels with a limited background. The generalizability of the index must be confirmed by further testing with a wider variety of foods and panels. To compute the similarity between trials, we used the Euclidean distance. Other popular distance measures include the Manhattan distance and distances based on probability. The effect of the choice of distance scale on the similarity index should be further studied. The similarity index developed in this study treats all attributes as having the same importance. However, it may be practical to use only essential attributes or to weight important attributes in order to compute similarity. Moreover, as demonstrated in this study, outlier analysis based on the developed similarity index can detect potentially outlying samples. However, it does not show how such samples differ from typical samples. Thus, a supplementary method may be desirable to help determine whether samples detected as outliers should be removed.

## 8. Conclusions

In the TDS method, the average of all trials is discussed generally, and few analyses of individual trials are conducted. Therefore, in this paper we have proposed a similarity index that can be used to compare individual TDS trials. This index can adjust the importance of the temporal similarity by changing the *R* parameter, which represents the number of discretizations. Using this index, we performed an outlier analysis for TDS on strawberry panel data. Different trials were classified as outliers with and without an emphasis on time series. These trials were confirmed to be vastly different from the average trial. Outlier analyses can be employed in all experiments using the TDS method to exclude outliers that substantially bias the average consumer responses. In addition, this index can be useful for other individual analyses that use distance measures, such as hierarchical clustering [42]. Challenges to be solved in the future include determining the appropriate *R* value for different analyses. The method used for outlier analysis can be improved in the future as well. Currently, it does not indicate the reasons behind certain samples being judged as outliers; thus, a method that is better able to explain these reasons is required.

## Figures and Tables

**Table 1:** Correlation coefficients $r_{R1,R2}$ with different R1 and R2 values. R1 and R2 are the numbers of discretization.

		R2					
		1	3	10	30	50	100
R1	1	1.000					
	3	0.797	1.000				
	10	0.675	0.826	1.000			
	30	0.521	0.594	0.848	1.000		
	50	0.385	0.429	0.693	0.941	1.000	
	100	0.251	0.269	0.548	0.865	0.972	1.000

**Table 2:** Detected outliers with different *R* values; + indicates outliers. Trial IDs consist of an alphabetic letter representing a panel and the iteration count of the trial.

		*R*					
		1	3	10	30	50	100
Trial ID	N1	+					
	E1			+	+		
	C2					+	
	C3	+	+	+	+	+	+

## Data Availability

Data is contained within the article.
